# Supplementary material for: Development of a computer-aided design software for the quantitative evaluation of aesthetic damage
Source: PLoS One. 2019 Dec 18;14(12):e0226322. doi: 10.1371/journal.pone.0226322 (PMC6919621; doi:10.1371/journal.pone.0226322)
Supplement: S1 Code site — 1. (ZIP) [file pone.0226322.s001.zip › [EESC_jr][Projeto]Relatorio_07_21/reports/3/2017.04.11/14:52:21/report.pdf]

Preâmbulo

Na data de 11/04/2017 no horário 15:00, o(a) Perito(a) Dr ROGERIO NOGUEIRA DE OLIVEIRA, Professor , designado(a)para proceder ao exame pericial solicitado em: Nelson Sakaguti, do sexo Masculino, nascido(a) em 11/04/1966, ocupação cirurgião-dentista, portador(a) do RG 151561 ssp/sp, cujo endereço RUA LINEU PRESTES, 2227, passa a expor a avaliação de dano estético corporal.

Exame objetivo da lesão

A lesão ora objeto de análise teve como natureza ocorrido em 01/01/2018. O prejuízo estético consiste em lesão cicatricial consolidada na região: Lábios, Pescoço no lado .

Trata-se de cicatriz Hipertrófica de tamanho/frequência Única: Retilínea de orientação Horizontal de pigmentação Normocrômica medindo 1 centímetro(s) na horizontal, 4 centímetros na vertical e 3 milímetros de Profundidade. De contornos Regulares. Perceptivel em 50 centímetros de distância nas relações íntimas. Perceptivel em 3 metros nas relações sociais. A lesão tem repercução na vida diária social, laboral conforme fotos anteriores e atuais..

Conclui-se que classificada segundo os parâmetros de dano estético AIPE - Análise do Impacto do Prejuízo Estético (escala de seis níveis e pontuação entre 0 e 50 pontos), como sendo dano estético relativo à área 1, a lesão é caracterizada por 21 pontos e grau de prejuízo estético: Importante.

Escore final: 21;
Grau de prejuízo: Importante.

FOUSP , 11/04/2017

____

Dr ROGERIO NOGUEIRA DE OLIVEIRA
